# Supplementary material for: Improving the Extraction of Catechins of Green Tea (Camellia sinensis) by Subcritical Water Extraction (SWE) Combined with Pulsed Electric Field (PEF) or Intense Pulsed Light (IPL) Pretreatment
Source: Foods. 2021 Dec 13;10(12):3092. doi: 10.3390/foods10123092 (PMC8701373; doi:10.3390/foods10123092)
Supplement: Supplementary file 1 [file foods-10-03092-s001.zip › foods-1493803-supplementary.pdf]

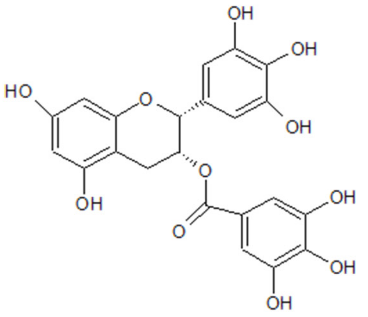

(-)-epigallocatechin gallate (EGCG)

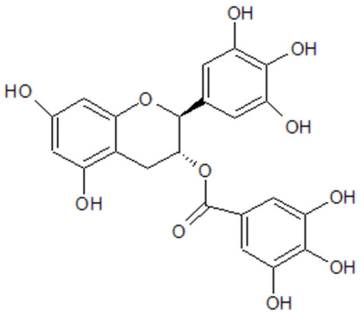

(-)-gallocatechin gallate (GCG)

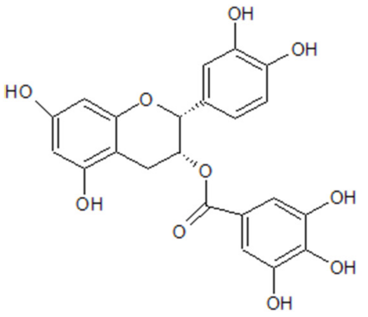

(-)-epicatechin gallate (ECG)

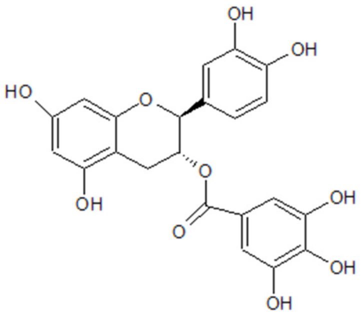

(-)-catechin gallate (CG)

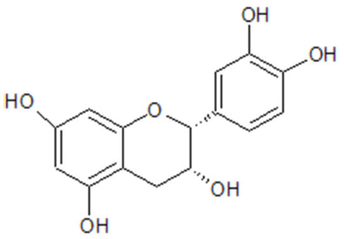

(-)-epicatechin (EC)

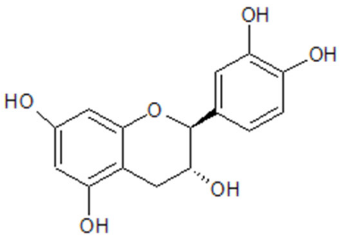

(-)-catechin (Ct)

Figure S1. Chemical structure of tea catechins.

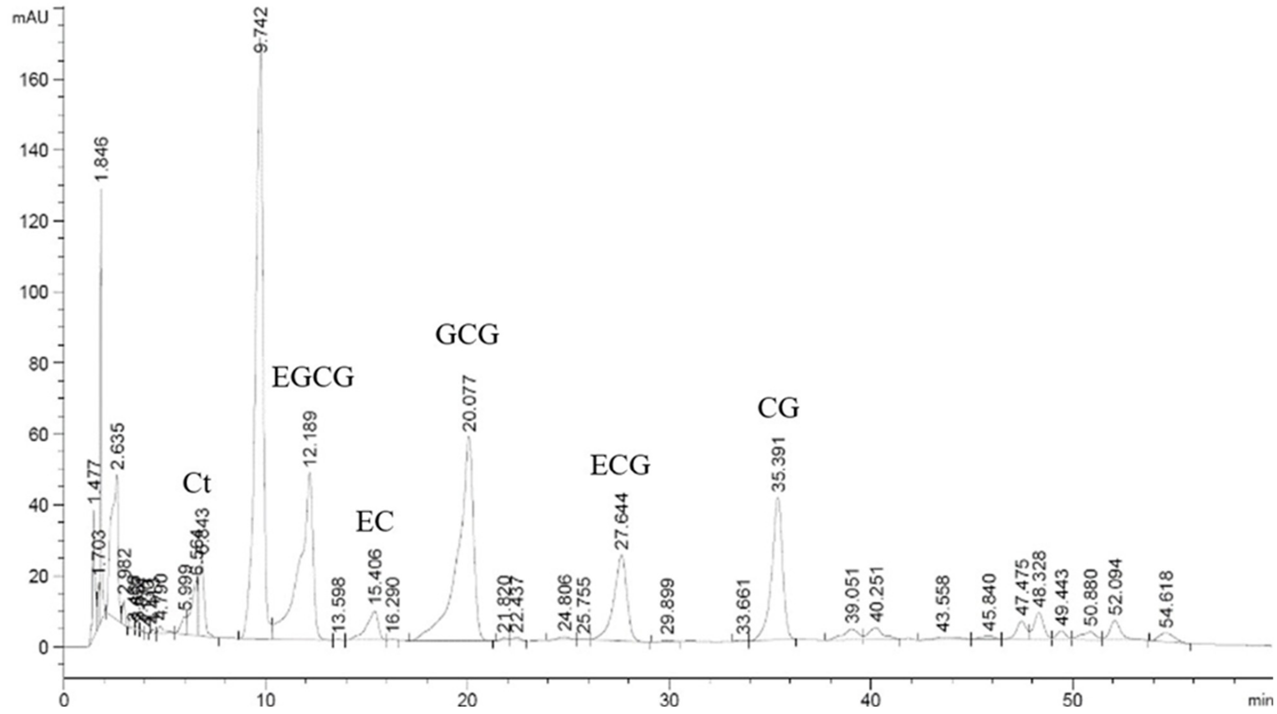

Figure S2. HPLC chromatogram of green tea leaves extract by SWE.
